# Phase dependent modulation of tremor amplitude in essential tremor through thalamic stimulation

**DOI:** 10.1093/brain/awt239

**Published:** 2013-10

**Authors:** Hayriye Cagnan, John-Stuart Brittain, Simon Little, Thomas Foltynie, Patricia Limousin, Ludvic Zrinzo, Marwan Hariz, Carole Joint, James Fitzgerald, Alexander L. Green, Tipu Aziz, Peter Brown

**Affiliations:** 1 Nuffield Department of Clinical Neurosciences, University of Oxford, John Radcliffe Hospital, West Wing Level 6, OX3 9DU, Oxford, UK; 2 Sobell Department of Motor Neuroscience and Movement Disorders, UCL Institute of Neurology, London, WC1N 3BG, UK; 3 Nuffield Department of Surgical Sciences, John Radcliffe Hospital, University of Oxford, Oxford, OX3 9DU, UK

**Keywords:** essential tremor, deep brain stimulation, thalamus, entrainment, plasticity

## Abstract

High frequency deep brain stimulation of the thalamus can help ameliorate severe essential tremor. Here we explore how the efficacy, efficiency and selectivity of thalamic deep brain stimulation might be improved in this condition. We started from the hypothesis that the effects of electrical stimulation on essential tremor may be phase dependent, and that, in particular, there are tremor phases at which stimuli preferentially lead to a reduction in the amplitude of tremor. The latter could be exploited to improve deep brain stimulation, particularly if tremor suppression could be reinforced by cumulative effects. Accordingly, we stimulated 10 patients with essential tremor and thalamic electrodes, while recording tremor amplitude and phase. Stimulation near the postural tremor frequency entrained tremor. Tremor amplitude was also modulated depending on the phase at which stimulation pulses were delivered in the tremor cycle. Stimuli in one half of the tremor cycle reduced median tremor amplitude by ∼10%, while those in the opposite half of the tremor cycle increased tremor amplitude by a similar amount. At optimal phase alignment tremor suppression reached 27%. Moreover, tremor amplitude showed a non-linear increase in the degree of suppression with successive stimuli; tremor suppression was increased threefold if a stimulus was preceded by four stimuli with a similar phase relationship with respect to the tremor, suggesting cumulative, possibly plastic, effects. The present results pave the way for a stimulation system that tracks tremor phase to control when deep brain stimulation pulses are delivered to treat essential tremor. This would allow treatment effects to be maximized by focussing stimulation on the optimal phase for suppression and by ensuring that this is repeated over many cycles so as to harness cumulative effects. Such a system might potentially achieve tremor control with far less power demand and greater specificity than current high frequency stimulation approaches, and may lower the risk for tolerance and rebound.

## Introduction

Essential tremor is a common movement disorder, for which effective non-invasive treatments remain limited (Deuschl and Elble, 2000). Despite its high prevalence rate, the pathophysiological mechanisms underlying and promoting essential tremor remain unclear. Nevertheless, the thalamus appears to play an important role, as evinced by the relative efficacy of high frequency deep brain stimulation (DBS) of the nucleus ventralis intermedius in suppressing essential tremor (Benabid *et al.*, 1991) and by studies highlighting tremor induction during low frequency stimulation of the thalamus (Hassler *et al.*, 1960; Constantoyannis *et al.*, 2004). In addition to altering tremor amplitude, ventrolateral thalamic stimulation may modulate tremor regularity; it has been reported that high frequency stimulation reduces the regularity of postural tremor (Vaillancourt *et al.*, 2003) whereas low frequency stimulation may pace essential tremor, although this observation has never been formally quantified (Bejjani *et al.*, 2000).

Further evidence for the key role of the ventrolateral thalamus in essential tremor pathophysiology is provided by the firing patterns exhibited by thalamic neurons in such patients. The discharges of these neurons are coherent with peripheral tremor (Hua and Lenz, 2005). Thalamic neurons also possess ion channel dynamics that can generate oscillations through inhibition-induced excitation (Jahnsen and Llinás, 1984*a*, *b*; Steriade *et al.*, 1990). The same neurons are involved in recurrent excitatory and inhibitory projections with cortical, inferior olive, brainstem and reticular neurons, which also express oscillation generating ion channels with appropriate synaptic time constants (Jahnsen and Llinás, 1984*a*, *b*; Sotelo *et al.*, 1986; Steriade *et al.*, 1990; Silva *et al.*, 1991; Wallenstein, 1994; Barman *et al.*, 1995; Sugihara *et al.*, 1995; Elble, 1996). These projections have the potential to further promote neural entrainment and contribute towards the emergence and reinforcement of a central tremor oscillator.

The above observations implicate a thalamic tremor oscillator that is either intrinsic to this nucleus, driven from outside or forms part of a more extensive tremor generating circuit. The response of thalamic oscillators to external perturbation could potentially clarify the role of the thalamus in essential tremor (Smeal *et al.*, 2010) and may provide insights into how tremor could be better controlled through DBS, in order to circumvent problems such as progressive tolerance to stimulation (Barbe *et al.*, 2011). Specifically, the rhythmicity and cellular properties of thalamic neurons raise the possibility that electrical stimulation delivered at particular instances in the tremor-generating cycle could differentially modulate tremor. In this study, we test the hypothesis that the effects of electrical stimulation on essential tremor can be phase dependent, and in particular seek evidence for a phase at which externally imposed perturbation preferentially leads to reduction in the amplitude of tremor. The latter could be exploited to increase the efficacy and efficiency of DBS, particularly if tremor amplitude suppression were cumulative, either through linear summation of stimulation effects or reinforcement by adaptive phenomena like spike-timing dependent plasticity (Tass and Majtanik, 2006; Popovych and Tass, 2012; Brittain *et al.*, 2013). Here, we use low-frequency electrical stimulation of the thalamus, closely matched with postural tremor frequency, to probe the nature of tremor in essential tremor and to define those tremor characteristics that might be targeted to increase stimulation efficacy.

## Materials and methods

### Patients and recordings

All patients gave their informed consent to take part in the study, which was approved by the local research ethics committee. Data from 10 patients with essential tremor were analysed to determine the effect of stimulation at near tremor frequency on essential tremor postural tremor. Patients had undergone unilateral or bilateral implantation of DBS electrodes into the ventrolateral thalamus for high frequency stimulation as a treatment of medically refractory essential tremor (Table 1). Techniques to target and implant electrodes in the ventrolateral thalamus have previously been described (Holl *et al.*, 2010). The permanent quadripolar macroelectrode used was model 3387 (Patients 1, 2, 3, 4, 5, 6 and 8) or model 3389 (Patients 7, 9 and 10) (Medtronic Neurologic Division) featuring four platinum-iridium cylindrical surfaces. Its contacts are numbered 0, 1, 2 and 3, with 0 being the most caudal and contact 3 being the most rostral. Localization was supported by the effect of intraoperative electrical stimulation and postoperative stereotactic CT or stereotactic MRI. Recordings were made from eight chronically implanted patients [i.e. >6 months following surgery: mean 1.5 + 0.4 (SEM) years], one patient 3–6 days following surgery and one patient 30 days after surgery. Tremor severity was evaluated in all patients preoperatively and in seven of the eight chronically implanted patients before conducting the study while DBS was set to the setting affording the best clinical outcome (Table 2). These evaluations were performed blinded to the electrophysiological results. On average, improvement in tremor severity was 12 ± 3.4 (SEM) points on the Bain and Findley tremor scale (Bain *et al.*, 1993). This represented a 70 ± 12% (SEM) improvement, supporting satisfactory DBS electrode placement.

**Table 1:** Clinical details

	Age	Most affected limb	Gender	Disease duration (years)	DBS contacts	Clinical DBS settings	Experimental DBS settings
1	59	RH	M	37	B+ 0−	3.6 V/90 µs/180 Hz	3.6 V/210 µs/ *f_T_* = 5 Hz
2	70	LH	M	52	B+ 1−	2.2 V/90 µs/130 Hz	2.2 V/240 µs/ *f_T_* = 7 Hz
3	67	LH	M	60	B+ 0−1−	2.5 V/60 µs/180 Hz	2.5 V/210 µs/ *f_T_* = 4 Hz
4	55	LH	M	35	B+ 0−	1.7 V/90 µs/130 Hz	1.7 V/210 µs/ *f_T_* = 5 Hz
5	71	RH	F	29	B+ 0−	3.5 V/90 µs/130 Hz	3.5 V/210 µs/ *f_T_* = 4 Hz
6	73	RH	M	7	B+ 1−	1.8 V/90 µs/180 Hz	1.5 V/240 µs/ *f_T_* = 6 Hz
7	61	LH	M	55	B+ 2−	2.7 V/60 µs/130 Hz	2.7 V/210 µs/ *f_T_* = 6 Hz
8	56	RH	M	38	1− 2+	2.5 V/90 µs/130 Hz	2.5 V/210 µs/ *f_T_* = 5 Hz
9	74	RH	M	28	B+ 2−	2.0 V/60 µs/130 Hz	2.0 V/210 µs/ *f_T_* = 4 Hz
10	34	RH	M	11	B+ 0−	1.8 V/60 µs/130 Hz	2.0 V/210 µs/ *f_T_* = 6 Hz

RH = right hand; LH = left hand; B = battery where stimulation is grounded to the implanted pulse generator.

**Table 2:** Preoperative and postoperative tremor severity scores

	R_pre_	P_pre_	K_pre_	I_pre_	T_pre_	R_post_	P_post_	K_post_	I_post_	T_post_	T_pre_ – T_post_
1	0	4	5	5	14	0	1	3	3	7	7
2	2	4	4	4	14	1	1	1	1	4	10
3	0	5	5	7	17	0	3	6	8	17	0
4	0	0	7	8	15	0	0	0	2	2	13
5	0	5	4	5	14	0	0	0	2	2	12
6	0	6	7	10	23	0	0	0	0	**0**	23
7	5	3	8	7	23	0	0	0	4	4	21
8	0	3	6	6	15	/	/	/	/	/	/
9	7	5	7	7	26	/	/	/	/	/	/
10	0	7	7	7	21	/	/	/	/	/	/

Scores are based on the Bain and Findley tremor severity scale for rest (R), postural (P), kinetic (K) and intention (I) components. T denotes the cumulative tremor score (i.e. R+P+K+I). Postoperative tremor severity has not been assessed for three patients (Patients 8–10), in two of whom 6 months had not elapsed from the date of surgery (Bain *et al.*, 1993). Pre and post refer to assessment timing with respect to surgery.

Silver/silver chloride EEG electrodes were placed over Cz and Fz and a tri-axial accelerometer (TMS International) was attached onto the index finger of the hand most affected by postural tremor. Accelerometer orientation was fixed across subjects. EEGs and the tri-axial accelerometer signal were recorded using a TMSI porti amplifier (TMS International) and custom written software. EEG was initially high-pass filtered at 0.5 Hz and both EEG and acceleration were low-pass filtered at 500 Hz. EEG and acceleration were sampled at 2048 Hz.

**Figure 1:**
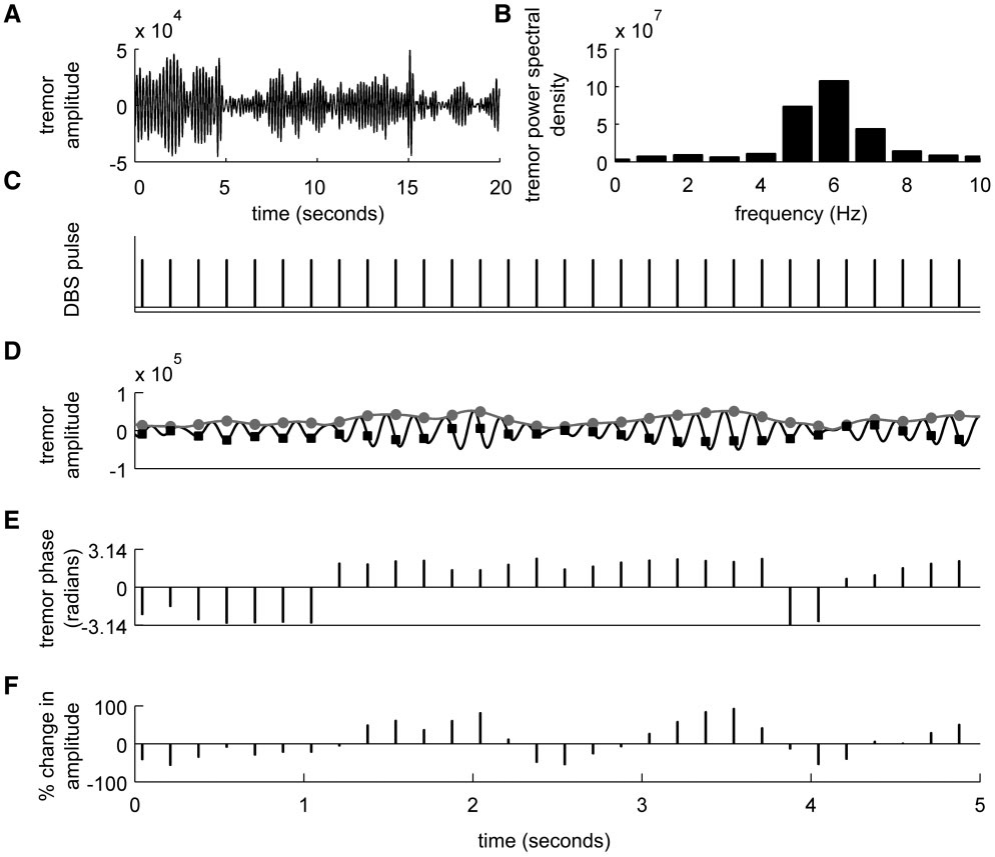
Summary of experimental protocol. (**A**) A short recording of the postural tremor was made while thalamic DBS was switched off and (**B**) power spectrum of the tremor signal was used to derive the tremor frequency. (**C**) Following the initial recording (**A**) to assess tremor frequency (**B**), stimulation pulses were applied at the nearest integer value of the tremor frequency (*f_T_* = 6 Hz). (**D**) Band-pass filtered tremor signal is shown in black and tremor envelope, which is derived from the Hilbert Transform of the tremor signal is indicated in grey during *f_T_* Hz stimulation. In order to construct the phase-amplitude profiles, we assessed changes in tremor envelope at the time of each stimulus (indicated as filled circles) and grouped these changes according to tremor phase at the same instance (indicated as filled boxes). (**E**) As stimulation frequency was not exactly matched to tremor frequency and due to spontaneous variations in tremor frequency, stimulation and tremor drifted in and out of phase, allowing stimulation pulses to coincide with different segments of the tremor cycle. (**F**) Percentage change in tremor envelope was derived with respect to the median tremor amplitude during each recording block, in order to dissociate natural fluctuations in tremor envelope from stimulation timing-dependent instantaneous modulations in tremor envelope. This formed the basis of phase-amplitude profiles.

Three blocks of recordings were made while subjects sat in a chair with their eyes open, with their most affected limb assuming a tremor provoking posture. The three blocks were with (i) DBS switched off; (ii) DBS set to the nearest integer frequency of the postural tremor frequency (*f_T_*); and (iii) DBS set to 2 Hz greater than postural tremor frequency (*f_T_*+2). Only the first two blocks of recordings could be acquired in Patients 4, 9 and 10. Stimulation parameters used during Blocks 2 and 3 are summarized in Table 1. After an initial recording to assess tremor frequency (Fig. 1A and B) and the posture that most consistently elicited tremor, the order of blocks was pseudorandomized between subjects. In six patients the most tremor-provoking upper limb posture consisted of holding their most affected limb outstretched in front, with the wrist slightly extended (Patients 1, 2, 4, 5, 6 and 10). In four patients tremor was more marked with the shoulder abducted, elbow flexed and wrist extended (Patients 3, 7, 8 and 9). To minimize fatigue, postures were maintained for 1 min, and followed by 30 s of rest before the arm was positioned again. On average 285 ± 14 s of recording were analysed for each block (i.e. DBS turned off or set to different frequencies). We only analysed time segments during which postural tremor was observed.

### Data analysis

Recordings were analysed offline using MATLAB®. Tri-axial accelerometer signals were band-pass filtered ±2 Hz around the postural tremor frequency using a fourth order Butterworth filter applied forwards and backwards. Tremor envelope (Fig. 1D) and instantaneous phase of the tri-axial accelerometer signals were estimated using the Hilbert Transform. Tremor amplitude envelope was derived using: 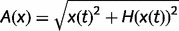, where *x(t)* is the band-pass filtered tremor signal and H(*x(t)*) is the Hilbert Transform of the band-pass filtered tremor signal. Instantaneous phase was obtained using: 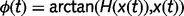. Instantaneous frequency was computed by differentiating the unwrapped phase.

EEG signals were high-pass filtered using a fourth order Butterworth filter with a 100 Hz cut-off frequency. This recovered stimulation artefact so that precise timing of each DBS pulse could be derived (Fig. 1C).

The effect of stimulation on the temporal characteristics of postural tremor was assessed using the instantaneous phase and amplitude envelopes of the band-pass filtered accelerometer signals whenever a DBS pulse was delivered in recording Blocks 2 and 3 (Fig. 1C–F). For recording Block 1 (i.e. DBS off), instantaneous phase and amplitude envelopes of the band-pass filtered accelerometer signals were sampled at the frequency of stimulation applied during either Block 2 or 3 for comparison purposes. Unless otherwise stated, we analysed the accelerometer channel that showed maximal change in tremor amplitude (i.e. maximal tremor amplitude range).

### Tremor entrainment

We analysed the degree of tremor entrainment across the entire stimulation block. Tremor phase sampled at the time instances when a DBS pulse was delivered (Fig. 1C–E) was divided into 21 phase bins of duration 0.3 radians. Tremor phase likelihood was derived by normalizing the number of elements in each phase bin by the total number of elements. Degree of tremor entrainment across the entire stimulation block was defined as the standard (z) score of the most likely phase value during stimulation with respect to the variability of tremor phase when DBS was turned off, sampled at the frequency of stimulation.

### Relationship between phase and amplitude

In order to investigate the relationship between the tremor phase at which each stimulation pulse was delivered and the changes in tremor envelope, we divided the percentage change in tremor envelope with respect to median tremor envelope amplitude observed during each recording block (Fig. 1F) into 21 bins depending on the corresponding tremor phase (Fig. 1E). Using a two-sided Wilcoxon rank-sum test at each phase bin (distributions were not normal; Kolmogorov-Smirnov test, *P* ≤ 0.05), we assessed whether the per cent change in amplitude-envelope during stimulation was significantly different from the amplitude-envelope variability observed when DBS was turned off. Results were corrected for multiple comparisons using the FDR procedure.

### Group phase-amplitude profile

**Figure 2:**
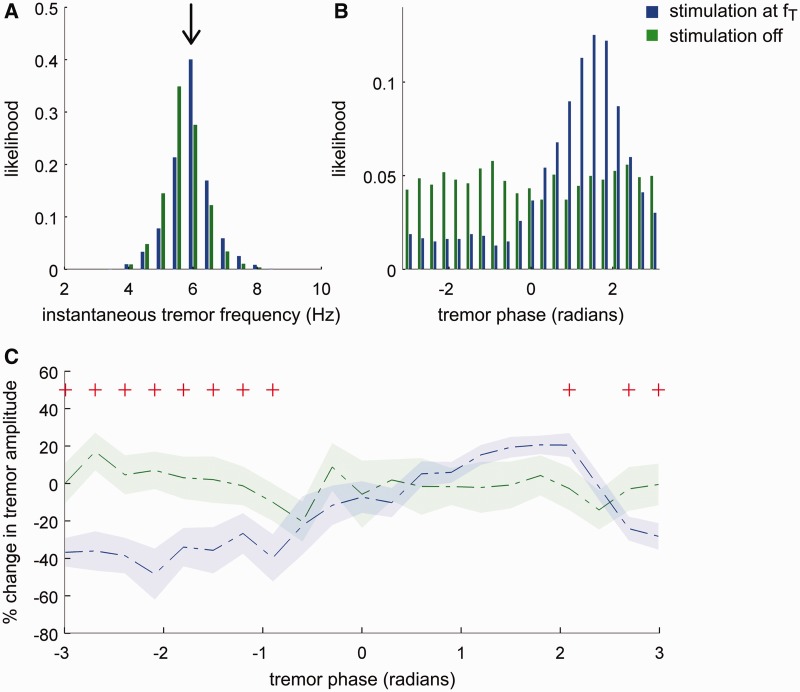
Exemplar effect of DBS on essential tremor in Patient 6. DBS at near postural tremor frequency (*f_T_*) alters various tremor properties, ranging from tremor frequency to tremor entrainment. Moreover, tremor amplitude is modulated differentially depending on the timing of the thalamic DBS pulses with respect to the tremor cycle. (**A**) Tremor frequency is altered due to stimulation at *f_T_* Hz and pulled towards stimulation frequency (*f_T_ = *6 Hz indicated with an arrow). (**B**) When DBS is off, tremor phase sampled at 6 Hz is uniformly distributed. During DBS at *f_T_* Hz (e.g. 6 Hz), tremor phase sampled at stimulation instances gets pulled to certain phases, indicating that DBS at *f_T_* Hz entrains postural tremor. (**C**) Median percentage change in tremor amplitude relative to corresponding tremor phase during *f_T_* Hz stimulation (phase-amplitude profile) and when DBS is switched off are indicated with dashed lines; while shaded regions indicate standard error of the mean (red plus sign indicates amplitude changes during *f_T_* Hz stimulation, which are significantly different from tremor amplitude variability when stimulation is switched off (two-sided Wilcoxon rank-sum test at each phase bin, FDR corrected for multiple comparisons). Thalamic stimulation at particular instances of the tremor cycle can attenuate or increase tremor amplitude.

Median tremor amplitudes observed at each phase bin (Fig. 2C; phase-amplitude profile for one patient; blue trace) were averaged across 10 patients in order to obtain the average phase-amplitude profile across all patients following alignment of each phase–amplitude profile so that 0 radians corresponded to either the phase value affording maximal tremor amplification, maximal tremor suppression or maximal entrainment. Average phase-amplitude profiles during stimulation at *f_T_* and *f_T_*+2 Hz were compared with corresponding average phase-amplitude profiles obtained from the DBS off block. The statistical significance of the percentage change in tremor amplitude due to a tremor pulse being delivered at a specific tremor phase was assessed using Student’s *t*-test (distributions were normal; Kolmogorov-Smirnov test, *P* > 0.05) between phase-amplitude profiles during stimulation at *f_T_* or *f_T_*+2 Hz and the corresponding phase-amplitude profiles obtained from the DBS off blocks sampled at either *f_T_* or *f_T_*+2 Hz. Significance levels were corrected for multiple comparisons using the FDR procedure.

### Consecutive stimuli at phase values favouring suppression or amplification

Individual phase-amplitude profiles revealed that there were certain phase values favouring suppression and certain phase values promoting amplification (Fig. 2C; phase-amplitude profile for one patient; blue trace). For each patient, the tremor phases at which stimulation pulses were delivered to the thalamus were separated according to whether stimulation at this phase value, on average, led to tremor suppression or tremor amplification. Percentage changes in the tremor amplitude envelope were grouped in bins 1 to 5 based on whether the corresponding phase value promoted on average suppression or amplification, and according to how many proceeding stimulation pulses favoured suppression or amplification (only one pulse timed as such, then bin 1, only two consecutive pulses timed as such then bin 2, etc). Due to on average 5% difference between the postural tremor frequency and stimulation frequency, the likelihood of consecutive stimuli having a similar phase relationship with respect to tremor decreased as the number of stimuli increased. Bins containing less than five instances per subject were disregarded in order to ensure a reliable average per bin per patient. Percentage change in tremor amplitude, offset by a fixed amount to ensure positive values, was log-normalized to ensure normality. The effect of consecutive stimuli at optimal phase was tested using repeated measures ANOVA with Greenhouse-Geisser correction for non-sphericity, where necessary. At least five instances of five consecutive stimuli at phase values favouring amplification were observed in eight patients, while for phase values favouring suppression, at least five instances of five consecutive stimuli were observed in nine patients. Therefore repeated measure ANOVAs were performed on data from eight patients for amplification and nine patients for suppression. *Post hoc* one-sided Student’s *t*-test between each block was corrected using FDR. The effects of consecutive stimuli on tremor suppression and amplification were fitted using a linear model (a+bx), quadratic model (a+bx+cx^2^) and power function (ax^b^+c) in order to investigate which functions best described these processes (Matlab®, Curve fitting toolbox).

## Results

Ten patients with essential tremor were stimulated at the nearest integer frequency (*f_T_*) of their tremor frequency. Stimulation was not actively locked to tremor through phase tracking of the tremor signal. Instead, stimulation and tremor were allowed to drift spontaneously in and out of phase (Fig. 1C–E). The effect of stimulation at *f_T_* is illustrated for one subject in Fig. 2. Postural tremor frequency was 5.5 Hz when DBS was switched off (Fig. 2A; depicted in green). During stimulation at *f_T_* (*f_T_* = 6 Hz), tremor frequency was pulled closer to stimulation frequency (Fig. 2A; depicted in blue) and tremor phase to a preferred phase region (Fig. 2B; depicted in blue). Degree of tremor entrainment across the entire stimulation block was derived from the standard (z) score of the most likely phase value during stimulation with respect to tremor phase variability when DBS was turned off. In the illustrated subject, standard score of the most likely phase value during stimulation (i.e. 1.5 radians) was 26 (Fig. 2B; depicted in blue). Thus DBS at *f_T_* tended to entrain tremor, so that the stimulus train and the tremor became more ‘in step’.

### Relationship between phase and amplitude

During DBS at *f_T_*, tremor amplitude was modulated differentially depending on the timing of stimulation pulses with respect to the tremor cycle (Fig. 2C; phase-amplitude profile for one patient; blue trace). In contrast, when DBS was switched off, tremor amplitude did not show any dependency on tremor phase when tremor phase was sampled at the same frequency (i.e. *f_T_*) (Fig. 2C, green trace). When DBS pulses were applied at an optimal tremor phase, in this specific example at −2 radians, tremor suppression could reach up to 48%, while applying DBS pulses at other regions of the tremor cycle either amplified tremor by up to 20% or kept tremor amplitude the same (Fig. 2C; phase-amplitude profile for one patient; blue trace). The per cent change in amplitude during stimulation was significantly different from amplitude variability observed when DBS was switched off (Fig. 2C).

In seven subjects, if a thalamic DBS pulse fell within −π to 0 radians of the tremor cycle, tremor suppression was promoted. Tremor phases at which stimulation promoted amplification, clustered over 0 to π radians. Thus, suppressive and amplifying effects of stimulation were anti-phase (i.e. separated by ∼180^o^). In three patients, there was no clear clustering of phase values according to induced amplitude effects. Two of these three subjects were recorded less than one month following their surgery, when data may have been affected by a postoperative stun effect. The third case (Tables 1 and 2; Patient 3) was a treatment failure, in whom there was no clinical effect of chronic high frequency DBS despite electrode location revision, during which appropriate surgical targeting of the ventrolateral thalamus was achieved and impedance testing that ruled out a circuit break (Blomstedt *et al.*, 2012).

If a DBS stimulation pulse was delivered to the thalamus at tremor suppression promoting regions of the tremor cycle (including optimal and non-optimal suppressive phases), on average −9.8 ± 2.1% tremor suppression was observed in 10 patients. Average tremor amplification at tremor amplification promoting regions of the tremor cycle on the other hand was 9.5 ± 2.2%.

### Group phase-amplitude profile

**Figure 3:**
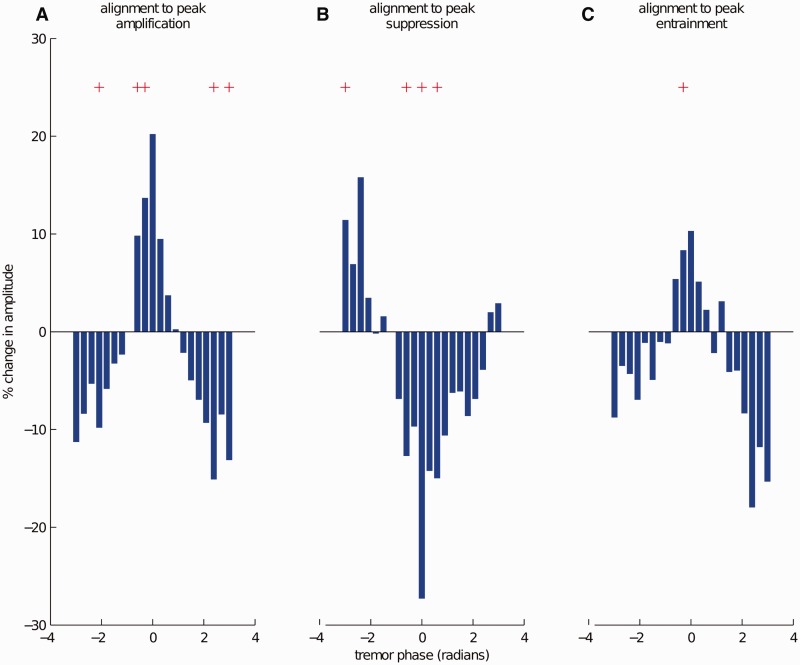
**Group data for amplitude changes due to stimulation at near postural tremor frequency (*f_T_*).** Individual phase-amplitude profiles were (**A**) aligned to peak amplification (at 0 radians); (**B**) aligned to peak suppression (at 0 radians); (**C**) aligned to the phase where maximal entrainment is observed (at 0 radians) and averaged in order to obtain the phase-amplitude profiles at the group level. Red plus sign indicates amplitude changes, which are significantly different from tremor amplitude variability when the stimulation is turned off (Student’s *t*-test at each phase bin, FDR corrected for multiple comparisons). Note that suppressive and amplifying effects were out of phase suggesting that stimulation might be interacting with an underlying, alternating, oscillatory pattern of neuronal excitability at tremor frequency.

Figure 3 shows the average dependence of tremor amplitude on stimulation phase (phase-amplitude profile) in 10 patients, when individual phase-amplitude profiles were aligned to the phase where maximal amplification was observed (Fig. 3A), maximal suppression was observed (Fig. 3B) or where peak entrainment was observed (Fig. 3C). Note that amplitude effects relate to the instantaneous amplitude of the tremor envelope estimated using the Hilbert Transform and not the instantaneous amplitude of the tremor signal (Fig. 1D). Tremor phases at which stimulation significantly modified tremor amplitude relative to the DBS-off state are indicated with red plus sign in Fig. 3. When phase-amplitude profiles were aligned to maximal amplification, both amplification and suppression were significant, again suggesting a relatively constant anti-phase relationship between amplification and suppression (Fig. 3A). The same held true when phase-amplitude profiles were aligned to maximal suppression (Fig. 3B). The evidence that suppressive and amplifying effects were 180^o^ out of phase suggests that stimulation might be interacting with an underlying, alternating, oscillatory pattern of neuronal excitability at tremor frequency. Across all subjects, on average we observed 27.3 ± 4.4% (mean ± SEM) maximal phase-aligned suppression of tremor amplitude and 20.2 ± 2.1% maximal phase-aligned amplification of tremor.

### Relationship between tremor entrainment and amplitude

**Figure 4:**
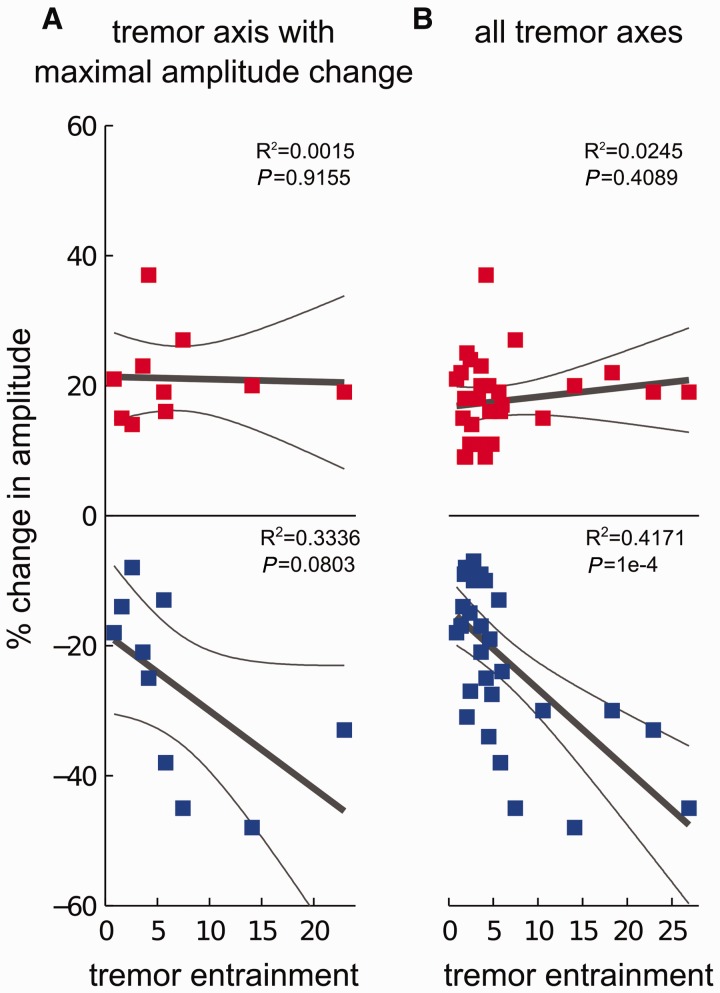
Relationship between tremor amplitude modulation during stimulation at near postural tremor frequency (*f_T_*) and the degree of tremor entrainment. Although tremor suppression was inversely proportional to tremor entrainment, tremor amplification did not show any dependency, possibly because tremor amplitude was already at ceiling values and could not be amplified further. Linear regression fits (thick grey lines) and their 95% confidence limits (thin grey lines) are shown in each panel. (**A**) Lower panel (in blue): tremor suppression showed a trend towards dependency on tremor entrainment at the tremor axis that showed maximal change in tremor amplitude (F-statistic *P* = 0.0803, R^2 ^= 0.3336). Upper panel (in red): in contrast, tremor amplification did not show any dependency on tremor entrainment at the same axis (F-statistic *P* = 0.9155, R^2 ^= 0.0015). (**B**) This difference between suppression and amplification was confirmed when changes across all three axes of the tri-axial accelerometer were considered. Lower panel: tremor suppression was inversely proportional to tremor entrainment (F-statistic *P* = 1 × 10^−4^, significant following FDR correction, R^2 ^= 0.4171), confirming the relationship between amplitude suppression and entrainment. Upper panel: tremor amplification did not show dependency on tremor entrainment (F-statistic *P* = 0.4089, R^2 ^= 0.0245).

Figure 4 shows the relationship between the degree of tremor entrainment and the maximal level of phase-aligned tremor suppression and amplification observed during stimulation at *f_T_*. The level of maximal tremor suppression is inversely proportional to the degree of tremor entrainment (Fig. 4A and B; blue), indicative of a relationship between tremor suppression and entrainment. Interestingly, the level of maximal tremor amplification was not dependent on tremor entrainment (Fig. 4A and B; red), perhaps because tremor amplitude was already at ceiling values.

### Relationship between clinical efficacy of deep brain stimulation and outcome of stimulating close to postural tremor frequency

**Figure 5:**
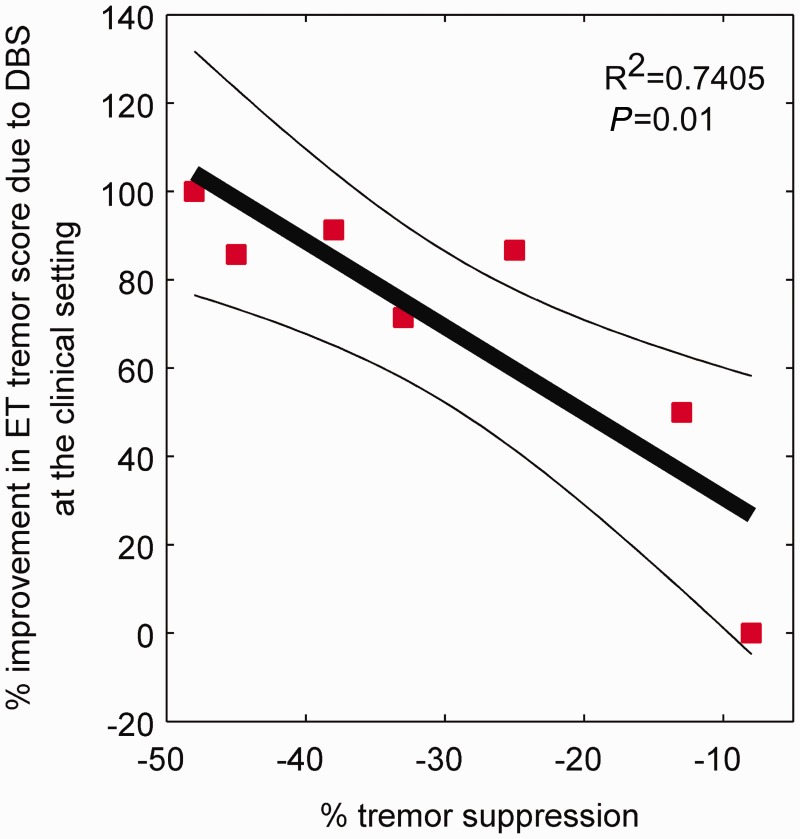
Correlation between clinical efficacy and effects of stimulation at near postural tremor frequency (*f_T_*). Relationship between maximal tremor suppression during DBS at *f_T_* Hz and per cent improvement in essential tremor (ET) severity during high frequency DBS with respect to tremor severity pre-DBS implantation. Clinical rating score was the Bain and Findley tremor rating score (Bain *et al.*, 1993). Linear regression fit is given by thick black line and its 95% confidence limits by thin black lines (F-statistic *P* = 0.01 and R^2^ statistic is 0.7405). This correlation suggests that clinical efficacy and the effects of stimulation at *f_T_* may share a common physiological basis.

Tremor severity could be clinically evaluated in seven patients preoperatively and again at least 6 months postoperatively while DBS was set to the parameters affording the best clinical outcome. Improvement in tremor severity ranged from 0 to 23 points on the Bain and Findley tremor scale [70 ± 12% (SEM)]. Maximal tremor suppression achieved during DBS at *f_T_* (Fig. 2C) was inversely proportional to the per cent improvement in tremor severity due to DBS at high frequency (*P* = 0.01; R^2 ^= 0.7405), suggesting that the two phenomena may share a common physiological basis (Fig. 5). Patient 3, who did not benefit from chronic high frequency DBS despite appropriate surgical targeting of the ventrolateral thalamus, also showed the least tremor suppression during DBS at *f_T_*.

### Effect of stimulation at other frequencies

**Figure 6:**
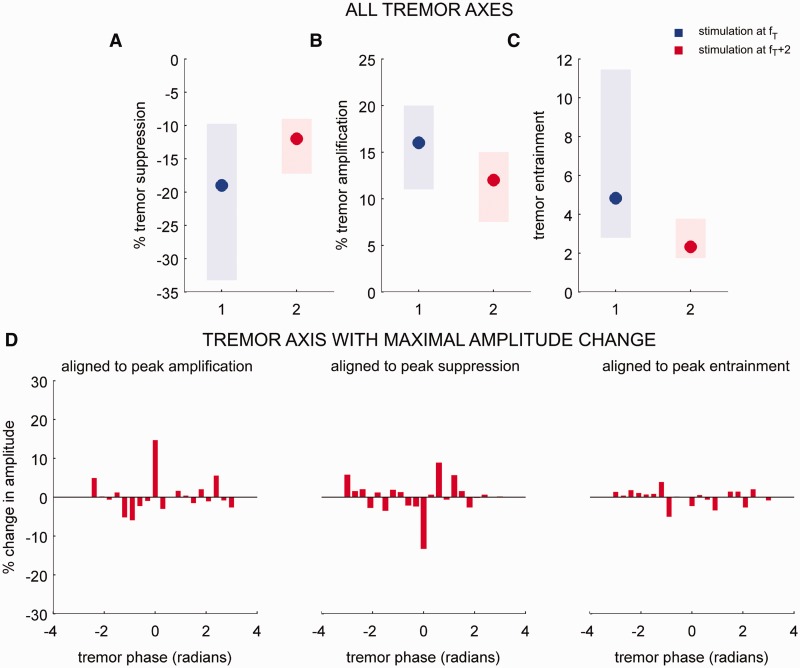
Characterizing the effect of *f_T_*+2 Hz stimulation on essential tremor. Tremor entrainment and the ability of stimuli at given phases to either suppress or amplify tremor amplitude were dependent on stimulation frequency. Shaded regions in **A−C** depict 25–75% percentiles and dots depict median values. (**A**) Group data for tremor suppression observed when thalamic stimuli coincided with the tremor phase affording maximal tremor suppression across all three tremor axes of the tri-axial accelerometer during *f_T_* Hz stimulation (blue) and *f_T_*+2 Hz stimulation (red). Tremor suppression observed during *f_T_*+2 Hz stimulation is significantly different from that observed during *f_T_* Hz stimulation (Wilcoxon rank sum test *P*-value = 0.0388, FDR corrected) (**B**) Same as **A** for tremor amplification observed when thalamic stimuli coincided with the tremor phase affording maximal tremor amplification (Wilcoxon rank sum test *P*-value = 0.0161, FDR corrected). Moreover (**C**) tremor entrainment was less than those observed during *f_T_* Hz stimulation (Wilcoxon rank sum test *P*-value = 0.0157, FDR corrected). (**D**) Group average phase-amplitude profile, observed during *f_T_*+2 Hz stimulation, at the tremor axis with maximal amplitude change. The amplitude profiles, when aligned to the phase where maximal amplification, suppression or entrainment was observed, showed no significant difference with respect to tremor amplitude variability when DBS was off. Phase-amplitude interactions for DBS off cases were reconstructed with respect to an artificial stimulation train at *f_T_*+2 Hz.

In order to assess whether the effects described above were stimulation frequency specific, DBS was also applied at *f_T_*+2 Hz. Maximal suppression (Fig. 6A) and maximal amplification (Fig. 6B) due to *f_T_*+2 Hz stimulation were significantly less than maximal suppression and maximal amplification observed during stimulation at *f_T_*. The degree of tremor entrainment observed during *f_T_*+2 Hz stimulation was also significantly less than that induced with *f_T_* Hz stimulation (Fig. 6C). Figure 6D shows the average phase-amplitude profile for the seven patients in whom stimulation at both *f_T_* and *f_T_*+2 Hz was performed aligned to the phase where maximal amplification, maximal suppression and peak entrainment were observed during *f_T_*+2 Hz stimulation. The average phase-amplitude profile during *f_T_*+2 Hz stimulation was not statistically different from that observed when DBS was off in contrast to those observed during *f_T_* Hz stimulation (Fig. 3). These results suggest that both the entrainment of tremor and the ability of stimuli delivered at certain phases of the tremor to modify tremor amplitude were dependent on stimulation frequency.

### Consecutive stimuli at phase values favouring suppression or amplification

**Figure 7:**
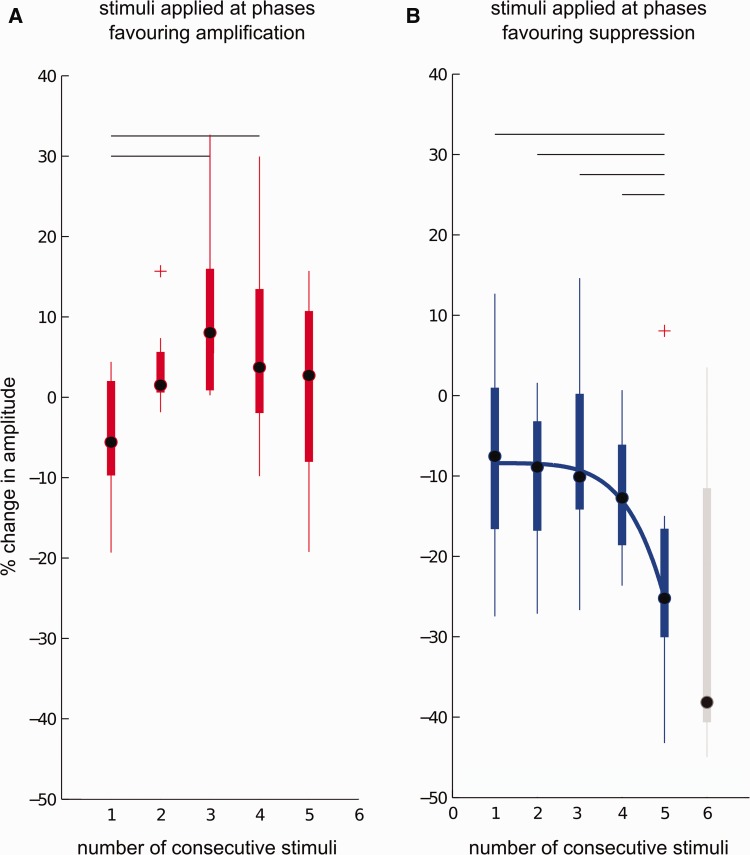
Effect of stimulation history on tremor amplitude during *f_T_* Hz stimulation. Tremor amplitude modulation shows a strong dependency on phase history and consistency of preceding pulses. Although tremor amplification plateaus and cannot be exacerbated, tremor suppression increases non-linearly with stimulation over consecutive cycles at tremor phases favouring suppression, indicating a cumulative suppressive effect of stimulation that can be exploited to increase tremor suppression during low frequency stimulation. (**A**) Group mean percentage change in tremor amplitude showed dependency on the number of consecutive stimuli delivered at tremor phase values favouring amplification (eight subjects, repeated measures ANOVA, *P* = 0.005). (**B**) Group mean percentage change in tremor amplitude showed dependency on the number of consecutive stimuli delivered at tremor phase values favouring suppression (nine subjects, repeated measures ANOVA, *P* = 0.02). Blocks of consecutive stimuli showing differences following multiple comparisons are highlighted with a horizontal line (10 multiple comparisons, *P* ≤ 0.05, FDR corrected). For suppression, the effect of consecutive stimuli on tremor amplitude was non-linear and was fitted with a power function, shown as a solid blue line (R^2 ^= 0.9919). If five thalamic pulses are consecutively delivered at tremor phases favouring suppression, tremor suppression increases threefold reaching a median suppression level of 26%. Effect of six consecutive stimuli is shown in grey. Median tremor suppression reaches 38%, indicating that the non-linear cumulative effect persists. As six consecutive stimuli were only observed in 50% of the subjects, this was not included in the repeated measures ANOVA or the fit. In **A−B**, the central dot is the median; edges of the boxes are the 25th and 75th percentiles while the whiskers extend to the most extreme data points not considered to be outliers. Outliers (denoted by a red cross) are plotted individually. A data point is classified as an outlier if it is outside q_25_ – 1.5*(q_75_ – q_25_) and q_75_ + 1.5*(q_75_ – q_25_) where q_75_ and q_25_ are the 75th and 25th percentile values.

Why should tremor phase-amplitude profiles be dependent on stimulation frequency? Due to differences between tremor frequency and *f_T_* and *f_T_*+2 Hz stimulation, in both cases, stimulation pulses drift in and out of phase with postural tremor. However, as *f_T_*+2 Hz is much faster than tremor frequency, stimulation at this frequency will drift in and out of phase with tremor a lot faster than stimulation at *f_T_*. Therefore, *f_T_* Hz stimulation is more likely to be associated with longer trains of consecutive stimuli with similar phase relationships to the ongoing tremor than stimulation at *f_T_*+2 Hz. Differential effects of stimulation at the two frequencies might have arisen if the phase history of preceding stimuli were to influence the effect of a pulse delivered at segments of the tremor cycle promoting suppression or reinforcement. In order to test this hypothesis, we grouped stimulation phase favouring suppression or amplification according to proceeding phase values and whether these also favoured suppression or amplification. Figure 7 shows the relationship between the number of consecutive stimuli delivered at phase values favouring amplification (red) or suppression (blue) and the percentage change in amplitude when DBS was applied at *f_T_* Hz. After five consecutive stimuli there was enhancement of the mean effect of stimulating at all tremor phases affording suppression from ∼10% to almost 30%. If the five patients in whom consecutive stimulation extended to six tremor cycles were considered, then median suppression continued to be further exaggerated and reached 38% (Fig. 7B). Given that tremor phase was not actively tracked in this study there were too few instances of more than six consecutive stimuli at tremor phases affording suppression to be analysed.

Two separate repeated measures ANOVAs confirmed that mean log-normalized percentage change in tremor amplitude was dependent on the number of consecutive stimuli delivered at tremor phase values favouring amplification (*P* = 0.005) or suppression (*P* = 0.02). *Post hoc* multiple comparisons are also shown in Fig. 7. The effect of consecutive stimuli at suppressive phase values (Fig. 7B) was quantitatively greater than those at amplifying phase values, which reached a plateau (Fig. 7A). Unfortunately, there were too few stimuli delivered at those tremor phases giving rise to maximal tremor suppression to estimate the combined effect of optimal phase precision and phase history. However, the present results strongly suggest that the phase and consistency of preceding stimuli is as important as the precise phase of a given pulse with respect to postural tremor phase.

The suppression profile in Fig. 7 was fitted with linear and non-linear functions (power function and quadratic model). Quadratic and power fits were both significant, however the power function provided a better fit with a *P-*value of 0.004 despite increased fit complexity and reduced degrees of freedom (R^2 ^= 0.9919). Linear regression of the suppression profile afforded a poorer fit and was not significant, confirming that the cumulative effect is non-linear (R^2 ^= 0.7511, *P* = 0.0572). The effect of consecutive stimuli on tremor amplification was also better represented using non-linear models (linear model R^2 ^= 0.3631; quadratic model R^2 ^= 0.8974; power function R^2 ^= 0.781), although fits obtained using both quadratic and power functions were not significant.

As also discussed below, the form of the power function, which describes the cumulative effect of consecutive stimuli delivered at phase values favouring suppression on tremor amplitude, raises the possibility of induction of short-term, spike-timing dependant plasticity. Accordingly we determined if the cumulative suppressive effect was sustained when the sixth pulse was delivered at a phase value now favouring amplification. The suppressive tendency persisted, with this pulse still inducing −19.4% suppression of tremor amplitude; significantly different to the effect of such a pulse presented at phases promoting amplification (two-sided Student’s *t*-test *P* = 5 × 10^−6^). Persistence of the suppressive tendency was, however, relatively short-lived, having dissipated ∼400 ms later if a seventh pulse also happened to be delivered at a phase value favouring amplification. This induced 4.6% suppression (i.e. not significantly different as compared to amplitude changes induced by stimulation at phases promoting amplification; two sided Student’s *t*-test, *P* = 0.1). In fact, persistence of the suppressive tendency of consecutive stimuli delivered at phase values favouring suppression on tremor amplitude also explains why the first value in Fig. 7A involves tremor suppression, despite delivery of the stimulus at a phase value favouring amplification. By definition the first pulse in this series is the first to follow a pulse or pulses at phase values favouring suppression.

## Discussion

We have shown that thalamic DBS at *f_T_* altered the temporal profile of the tremor observed in patients with medically refractory essential tremor. Temporal changes took the form of tremor entrainment, and tremor phase-dependent suppression and amplification of tremor. Tremor phase-dependent amplitude modulation of essential tremor has not been previously reported and was significantly different from the spontaneous tremor variation observed when DBS was switched off. The change in tremor amplitude averaged across all stimulation phases promoting suppression and all phases promoting amplification was just under −10% and 10%, respectively. This increased to −27% and +20% if tremor amplitude changes were assessed at the optimum tremor phases for the corresponding effects.

The level of suppression that could be achieved at specific tremor phases correlated with the degree of improvement in essential tremor severity during therapeutic high frequency DBS relative to tremor severity pre-DBS implantation. This suggests that the two phenomena may share a common physiological basis. It has been previously reported that the efficacy of DBS decreases with decreasing DBS frequency. Several experimental and theoretical papers have addressed this dependency (Benabid *et al.*, 1991; Gao *et al.*, 1999; Rizzone *et al.*, 2001; Moro *et al.*, 2002; Rubin and Terman, 2004; Cagnan *et al.*, 2009). One possible explanation for the relationship between DBS efficacy and DBS frequency was provided by a theoretical model where it was hypothesized that higher DBS frequencies allowed for an increased probability of stimulating the underlying pathological oscillation at the right time and disrupting relay of this oscillation to cortex (Cagnan *et al.*, 2009). The present results with thalamic stimulation at *f_T_* confirm the predictions of the theoretical model in so far as tremor amplitude suppression depends on the specific phase of the tremor cycle at which stimulation pulses are delivered.

### Cumulative effects during low frequency deep brain stimulation

Our effects on the temporal profile of tremor were frequency selective. Phase dependent modulation of tremor amplitude was, in particular, limited to stimulation at tremor frequency and was lost if stimulation frequency was offset by 2 Hz. This observation is interesting. Clearly, with both stimulation at *f_T_* and *f_T_*+2 Hz, some pulses hit the tremor cycle at the optimal phases for suppression and reinforcement, and yet a significant tremor amplitude modulation was only seen during stimulation at *f_T_*. Due to differences between tremor frequency and stimulation frequency, during stimulation at *f_T_* or *f_T_*+2 Hz, stimulation pulses would drift in and out of phase with postural tremor. Stimulation at *f_T_* is more likely to be associated with longer trains of consecutive stimuli with similar phase relationships to the ongoing tremor than stimulation at *f_T_*+2 Hz as *f_T_* Hz is closer to tremor frequency; stimulation at this frequency will drift in and out of phase with tremor a lot slower than stimulation at *f_T_*+2 Hz. Thus the differential effects of stimulation at the two frequencies might arise if the phase history of preceding stimuli were to influence the effect of a pulse delivered at the optimal phase for suppression or reinforcement. This could arise through the accumulation of the effects of previous stimuli, if individually these last more than one tremor cycle, or through spike-timing dependent adaptive processes. For both tremor suppression and tremor amplification, the effect of consecutive stimuli on tremor amplitude was non-linear. The importance of the phase history of preceding stimuli was borne out, and was particularly marked in the case of tremor suppression; when stimulation happened to be applied at phase values favouring tremor suppression over five cycles, the mean effect across all suppressive phases was increased ∼threefold, from <10% to ∼30%. Thus prominent tremor suppression required stimulation to be delivered at phases promoting suppression over several tremor cycles, which happens with higher probability during stimulation at *f_T_*. Accordingly, there was also a striking correlation between tremor amplitude suppression and greater tremor entrainment during stimulation at *f_T_*. Both these processes will act to increase the number of consecutive cycles when stimulation may land at the critical phase point. The implication is that tremor suppression involves a cumulative effect, which judging by the form of the power function that best fit the data, was unlikely to be due to the linear summation of any persistent suppressive effects of previous stimuli. The latter would give a function that involved an initial steep increase in suppression that then plateaus; the opposite of the power function that best fitted the data. The cumulative effect might instead potentially be mediated by short-term, spike-timing dependent plasticity (see below), consistent with the short-lived persistence of the suppressive effect. Alternatively, it might arise through entrainment of one oscillator in a system of multiple oscillators; entrainment and hence, in effect isolation of one oscillator, will take time to establish and likewise time to disestablish.

A cumulative effect was also evident in the case of tremor amplification following consecutive stimuli at tremor phases promoting amplification. However, this was much less marked than with tremor suppression, perhaps due to a ceiling effect limiting further tremor amplification in patients in whom tremor was already marked; but see below for another possible explanation. The relative weakness of this cumulative amplification effect, which remained at <10% increase, might also explain why there was little correlation between tremor amplitude reinforcement and greater tremor entrainment during stimulation at *f_T_*.

### Phase dependency of the effects of low frequency stimulation

But why should there be critical phases for tremor amplitude modulation and why should the effect depend on the consistency with which this phase is hit by stimulation over consecutive cycles? One clue is that the critical phases for amplitude suppression and amplification were 180° out. This suggests that stimulation is interacting with an underlying, alternating, sinusoidal pattern of neuronal excitability at tremor frequency. Such a pattern of oscillatory synchronized neuronal activity is consistent with the coherence between the firing patterns of thalamic neurons and tremor in essential tremor (Hua *et al.*, 1998; Hua and Lenz, 2005), something that is also seen in Parkinson’s disease tremor (Lenz *et al.*, 1988, 1994; Brodkey *et al.*, 2004). Similarly, oscillations have been detected in the thalamic local field potential that are coherent across sites within ventralis intermedius, and ventralis oralis posterior, and are coupled to peripheral tremor in essential tremor (Kane *et al.*, 2009). However, other brain regions are also implicated in tremor generation such as the cortex, cerebellum, brainstem and the basal ganglia (Hellwig *et al.*, 2001). Thus, essential tremor may be generated by a complex synchronized network, emerging from the coupling across multiple sites. When a pulse is delivered to a neural oscillator, such as the thalamocortical neuron, spike timings can be phase advanced or phase delayed depending on the phase of the pulse (Hansel *et al.*, 1995; Ermentrout, 1996; Smeal *et al.*, 2010). The timing of pre- and post-synaptic action potentials with respect to each other determines the strength of the connection between two neurons (Markram *et al.*, 1997; Bi and Poo, 1998; Egger *et al.*, 1999; Nishiyama *et al.*, 2000; Froemke and Dan, 2002; Zucker and Regehr, 2002). One possible explanation for the phase dependency of tremor suppression and amplification is that DBS pulses delivered at the ventrolateral thalamus could potentially phase advance or phase delay the spike timings of the thalamocortical relay neurons, depending on the phase of stimulation with respect to the ongoing oscillation. This would in turn enhance or reduce the efficacy of each thalamocortical spike, hence, temporarily enhancing or reducing the efficacy of synaptic connections with thalamocortical neurons. Given the evidence that thalamocortical neurons are in a highly synchronized state in essential tremor (Kane *et al.*, 2009), the possibility of enhancement may be limited because synaptic strength between neurons may already be high. This could explain the plateau observed in tremor amplification by consecutive stimuli at optimal phase alignment for amplification and the lack of dependency of tremor amplification on tremor entrainment.

A broadly similar dependency of amplitude effects on tremor phase has been reported with transcranial alternating current stimulation of the motor cortex in patients with Parkinson’s disease, where it has been proposed that sinusoidal current acts, through linear summation, to damp spontaneous tremor-related oscillatory activity (Brittain *et al.*, 2013). By stimulating at the critical phase point for 30 s, Brittain *et al.* (2013) increased the scale of tremor suppression by up to 50%. Although we submit that the mechanism involved here is not simple phase cancellation, the present results suggest that similar adaptive effects might be had in essential tremor and, moreover, that these can be secured with very brief biphasic pulses of electrical stimulation.

Our approach should be distinguished from that of coordinated reset neuromodulation. The latter uses the phase-resetting properties of a stimulus (single pulse or high frequency pulse train) in order to decouple populations of locally synchronized neurons. Phase-resetting of these neural populations, which are presumed to be spatially distributed within the target nucleus, is accomplished by applying pulses through different DBS electrode contacts at different times (Tass, 2003; Tass *et al.*, 2012). Two features set this approach apart from that taken here. First, coordinated reset neuromodulation requires spatially patterned stimulation across different contacts of the DBS electrode. Second, coordinated reset neuromodulation can be applied open-loop without specifying the phase relationship between the stimulation and the underlying oscillations (Popovych and Tass, 2012). The potential therapeutic effects of stimulation at particular tremor phases, as presented here, will depend on phase tracking so that stimulation will need to be delivered in a closed-loop mode. Nevertheless, there are broad similarities between the two approaches. First, although no attempt is made to fracture local synchronization in the current study, we may still be decoupling the thalamocortical population from other tremor-generating oscillators within the larger tremor circuit through the phase-resetting properties of DBS pulses. Second, the therapeutic potential of both techniques may possibly be promoted through the engagement of plasticity. In particular, a recent study in a non-human primate model of Parkinson’s disease has suggested that coordinated reset neuromodulation can have pronounced and long-term plastic effects (Tass *et al.*, 2012).

### Essential tremor pathophysiology

Perturbing the thalamic oscillator at frequencies close to the peripheral tremor frequency modulated temporal characteristics in a phase-dependent fashion implying that the thalamus is not merely a passive relay nucleus in the tremor-generating mechanism (Hansel *et al.*, 1995; Ermentrout, 1996; Smeal *et al.*, 2010). Intriguingly, tremor modulation was only observed when stimulation frequency was close to that of the underlying tremor; stimulation of the thalamus with a 2 Hz frequency offset was not effective in modulating or entraining the tremor-generating oscillator. Hence, stimulation intensity at *f_T_*+2 Hz was below the critical value for entrainment, even though we stimulated at the same intensity, which afforded therapeutic effects, and used a long pulse duration (≥210 µs). The critical stimulation intensity for entrainment varies in proportion with the strength of the coupling between oscillators in a network (Antonsen *et al.*, 2008). Therefore, the lack of entrainment or instantaneous frequency changes during strong stimulation at *f_T_*+2 Hz suggests that thalamic stimulation is interacting with a tremor circuit that may involve multiple strongly coupled oscillators.

### Implications for therapy

The degree of instantaneous tremor suppression achieved in the present study fell short of a clinically useful effect. However, our results suggest that marked amplitude effects could be achieved if tremor phases were tracked so that stimuli could be consistently delivered at the optimal suppressive phase with respect to on-going tremor. This arises because of two data features; first, within the region of the tremor cycle affording tremor suppression, there were optimal phases when stimuli could elicit almost 30% suppression, regardless of the phase history of preceding pulses. Phase tracking could ensure consistent stimulation at precisely these optimal phases. In contrast, we observed on average 10% tremor suppression when stimuli were less acutely timed within the region of the tremor cycle favouring suppression. As in this study we used the natural drift between two rhythmic processes at similar frequencies, we were not able to track the effect of consecutive stimuli at the very optimal phase for suppression. However, we were able to assess the effect of up to six consecutive stimuli with less acute timing within the region of the tremor cycle favouring suppression. This revealed the second feature suggesting that tremor suppression might be clinically significant if tremor were tracked so that stimuli could be consistently delivered according to tremor phase. This was the steep increase in suppression with increasing numbers of consecutive stimuli at suppressive phases, even when stimuli were less precisely timed within the region of the tremor cycle favouring suppression.

Thus the present results raise the exciting prospect that clinically useful tremor suppression could be achieved with phase-controlled low frequency stimulation of the thalamus. This would have the advantage that power demands would be much lower than with current high frequency stimulation and specificity would also potentially be improved. Such specificity emerges as a natural consequence of the dependency of the effects of low frequency stimulation on their delivery at critical points in the phase of the oscillations underlying the pathological tremor; physiological patterns of synchronization would not be expected to share this critical phase locking to stimulation and should be relatively unaffected. Furthermore, a stimulation approach that promotes cumulative suppressive plastic effects might offer a means of overcoming the tolerance to prolonged high frequency DBS that sometimes develops with stimulation in essential tremor (Barbe *et al.*, 2011). Our results suggest that low frequency stimulation at a specific phase relationship with on-going tremor oscillations could interact with the underlying oscillatory network, possibly resetting its intrinsic functional connectivity in a way that does not seem to happen with high frequency DBS in essential tremor. In the latter case stimulation is at too high a frequency to enlist entrainment and to take advantage of cumulative phase-dependent effects, and the response to stimulation over time may even wane through adaptation of the biological response of the neuronal network stimulated at these high frequencies (Barbe *et al.*, 2011).

Tremor suppression using active phase tracking to deliver electrical current to the motor cortex at the optimal phase for suppression has been piloted in Parkinson’s disease (Brittain *et al.*, 2013). The present findings suggest that active phase tracking could also be employed to control when DBS pulses are delivered to control essential tremor. This would allow treatment effects to be maximized by focussing stimulation on the optimal phase for suppression and by ensuring that this is repeated over many cycles to capture cumulative effects. The current results provide an important impetus for trials of closed-loop phase tracking stimulation regimens in essential tremor.

## Funding

This work was funded by the Medical Research Council and the National Institute of Health Research, Oxford Biomedical Research Centre.

## Abbreviations

DBS: deep brain stimulation
